# Selective Pressure for Biofilm Formation in Bacillus subtilis: Differential Effect of Mutations in the Master Regulator SinR on Bistability

**DOI:** 10.1128/mBio.01464-18

**Published:** 2018-09-04

**Authors:** Jan Kampf, Jan Gerwig, Kerstin Kruse, Robert Cleverley, Miriam Dormeyer, Alexander Grünberger, Dietrich Kohlheyer, Fabian M. Commichau, Richard J. Lewis, Jörg Stülke

**Affiliations:** a Department of General Microbiology, Georg-August-University Göttingen, GZMB, Göttingen, Germany; b Institute for Cell and Molecular Biosciences, Newcastle University, Newcastle upon Tyne, United Kingdom; c Forschungszentrum Jülich GmbH, IBG-1: Biotechnology, Jülich, Germany; d Aachener Verfahrenstechnik (AVT.MSB), RWTH Aachen University, Aachen, Germany; University of Illinois at Chicago

**Keywords:** biofilm formation, bistability, gene expression, microfluidics, suppressor mutants

## Abstract

Biofilm formation by Bacillus subtilis requires the expression of genes encoding enzymes for extracellular polysaccharide synthesis and for an amyloid-like protein. The master regulator SinR represses all the corresponding genes, and repression of these key biofilm genes is lifted when SinR interacts with its cognate antagonist proteins. The YmdB phosphodiesterase is a recently discovered factor that is involved in the control of SinR activity: cells lacking YmdB exhibit hyperactive SinR and are unable to relieve the repression of the biofilm genes. In this study, we have examined the dynamics of gene expression patterns in wild-type and *ymdB* mutant cells by microfluidic analysis coupled to time-lapse microscopy. Our results confirm the bistable expression pattern for motility and biofilm genes in the wild-type strain and the loss of biofilm gene expression in the mutant. Moreover, we demonstrated dynamic behavior in subpopulations of the wild-type strain that is characterized by switches in sets of the expressed genes. In order to gain further insights into the role of YmdB, we isolated a set of spontaneous suppressor mutants derived from *ymdB* mutants that had regained the ability to form complex colonies and biofilms. Interestingly, all of the mutations affected SinR. In some mutants, large genomic regions encompassing *sinR* were deleted, whereas others had alleles encoding SinR variants. Functional and biochemical studies with these SinR variants revealed how these proteins allowed biofilm gene expression in the *ymdB* mutant strains.

## Abstract (precis)

Many bacteria are able to choose between two mutually exclusive lifestyles: biofilm formation and motility. In the model bacterium Bacillus subtilis, this choice is made by each individual cell rather than at the population level. The transcriptional repressor SinR is the master regulator in this decision-making process. The regulation of SinR activity involves complex control of its own expression and of its interaction with antagonist proteins. We show that the YmdB phosphodiesterase is required to allow the expression of SinR-repressed genes in a subpopulation of cells and that such subpopulations can switch between different SinR activity states. Suppressor analyses revealed that *ymdB* mutants readily acquire mutations affecting SinR, thus restoring biofilm formation. These findings suggest that B. subtilis cells experience selective pressure to form the extracellular matrix that is characteristic of biofilms and that YmdB is required for the homeostasis of SinR and/or its antagonists.

## INTRODUCTION

Free-living bacteria possess a large variety of sensing and regulatory systems that allow an appropriate response to the rapidly changing environmental conditions that any cell may encounter. Such responses consist of two different and intensively studied layers: the adaptation of metabolism and the control of gene expression. Recently, it has become clear that the acquisition of mutations that provide a selective growth advantage, when the routine regulatory programs do not help, is a third layer of adaptation. This genomic adaptation has been observed when bacteria leave their “comfort zone” of metabolic homeostasis under conditions of extreme limitation of essential ions, metabolite imbalance, or conditions of osmotic pressure (1–7). Bacteria may also adapt to the commonly encountered environmental alterations in a completely different way; genetically identical populations can form distinct subpopulations with different physiological properties to allow continued growth of some of the population when many cells cannot grow. This growth strategy is referred to as bet hedging (8). Specific molecular switches that are active in only a part of the population form the molecular basis of bet hedging. These bistable switches often depend on threshold concentrations of stimulatory ligands to become active, which can be achieved only stochastically in some cells (8, 9).

In the Gram-positive soil-dwelling bacterium Bacillus subtilis, bistable gene expression has been studied intensively in biofilm formation, motility, sporulation, and spore killing (8, 10). In biofilm formation and motility, the activity of a small DNA-binding transcription factor, SinR, is central for the determination of cell fate (11). SinR forms tetramers and binds to operator sites in the promoter regions of the major operons for biofilm formation, namely, *epsA-O* and *tapA-sipW-tasA* (11–13). These operons are required for extracellular polysaccharide synthesis and for production and proper deposition of amyloid fibers in the extracellular matrix, respectively (14, 15). The activity of SinR is controlled by two antagonist proteins, SinI and SlrR. Binding of either SinI or SlrR to SinR inhibits the latter’s DNA-binding capacity and relieves the biofilm operons from repression, resulting in the formation of a biofilm or a pellicle structure in liquid (12, 13, 16). Excess SinR represses the biofilm operons if SinR levels exceed those of its antagonists (17). The expression and subsequent accumulation of the antagonists are controlled by a variety of transcription factors, including SinR and the master regulator of sporulation and differentiation initiation, Spo0A (18). However, the environmental cues that govern the expression and activity of SinR and its interaction partners remain poorly understood.

The complex interactions between SinR and its antagonists result in bistability; i.e., each cell in a population can express either the genes for biofilm formation or the genes for motility but not both (11). The mutual exclusivity of these two physiological states is achieved not only by controlling the expression of biofilm and motility genes but also by a clutch-like interaction of EpsE with the FliG motor to prevent motility once biofilm gene expression is activated (19).

The YmdB phosphodiesterase is involved in controlling the bistable switch between biofilm and motility gene expression (20). Biofilm genes are not expressed in the absence of YmdB, whereas all the SigD-dependent genes required for motility and cell chain separation are highly expressed in a *ymdB* mutant (21). YmdB degrades cyclic nucleotide monophosphates *in vitro* (21) but the physiological substrate of YmdB has not yet been discovered. Importantly, the enzymatic activity of YmdB is crucial for the control of bistability, suggesting that YmdB degrades or converts a so-far-unidentified substrate (21). YmdB may act in the signaling chain upstream of SinR because inactivation of SinR or overexpression of the SlrR antagonist can overcome the lack of biofilm formation in a *ymdB* mutant. YmdB has also been implicated recently in sporulation, in nanotube formation for the exchange of molecules between individual cells, and in colony development (22–25), but its precise role in these aspects of bacterial physiology has yet to be elucidated.

In this study, we addressed bistable gene expression of biofilm and motility genes in single living cells using a microfluidic platform to observe the switch of individual cells from one gene expression program to the other. We isolated and characterized a large set of suppressor mutants to gain more insights into the direct targets of YmdB that may in turn control the state of SinR. All mutants affected *sinR*, suggesting that YmdB controls SinR expression and/or activity. A biochemical analysis of several mutant SinR proteins provided a molecular explanation of the effects of the mutations on the interaction between SinR and its antagonist SinI and its DNA target.

## RESULTS

### Phenotypic heterogeneity of B. subtilis wild-type and *ymdB* mutant strains.

**FIG 1:**
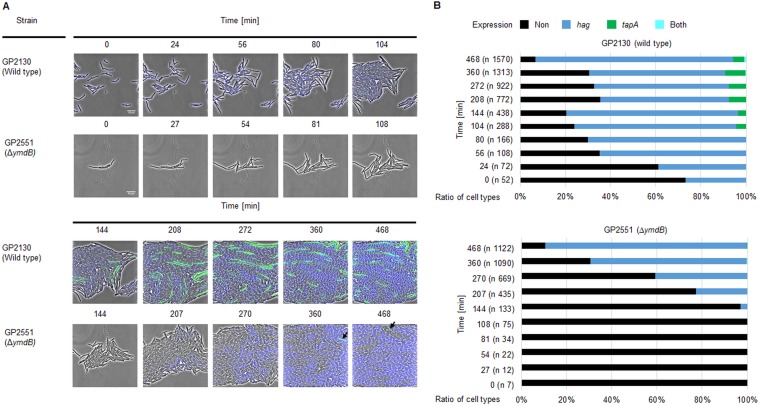
Microfluidic single-cell cultivation and analysis of B. subtilis. (A) Time-lapse image series of B. subtilis cells harboring transcriptional fusions of P_*hag*_-*cfp* (motility genes) and P_*tapA*_-*yfp* (biofilm genes). Wild-type cells (GP2130) and isogenic Δ*ymdB* mutant cells (GP2551) grown in LB medium at 37°C in microfluidic chambers are shown in the upper and lower panels, respectively, as indicated. Black arrows indicate the appearance of suppressor mutants that regained the ability to express biofilm genes. (B) Ratio of cell types at each time point of the time-lapse image series. n, total number of cells whose expression was determined at each time point.

Our previous work has shown that YmdB is required for bistable expression of motility and biofilm genes in B. subtilis (20). However, heterogeneous gene expression of motility and biofilm genes in real time has not been reported before for growing cells of B. subtilis. Time-lapse fluorescence microscopy was thus used to visualize the expression of motility (*hag*) and biofilm (*tapA*) genes in single cells cultivated in a microfluidic chamber to obtain new insights into bistability dynamics. A strain was thus constructed with ectopic transcriptional fusions of the genes for cyan fluorescent protein (*cfp*) and yellow fluorescent protein (*yfp*) to the *hag* and *tapA* promoters, respectively. The gene encoding flagellin (*hag*) was deleted to prevent movement of bacteria inside the microfluidic chamber. The fluorescence of cells of the wild-type strain (GP2130) and the isogenic *ymdB* mutant (GP2551) was recorded, and the distribution of the different cell types was analyzed (Fig. 1).

**FIG 2:**
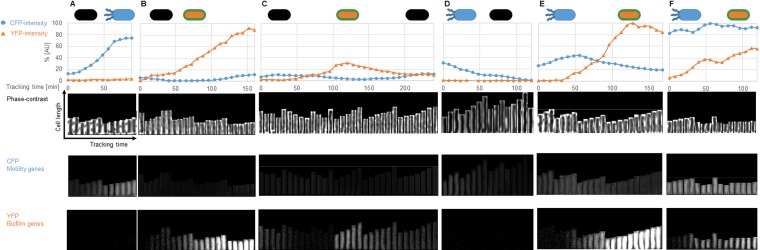
Tracking of single cells during microfluidic single-cell cultivation of B. subtilis wild type. Single-cell tracking of B. subtilis wild-type cells (GP2130) carrying transcriptional fusions of P_*hag*_-*cfp* (motility genes) and P_*tapA*_-*yfp* (biofilm genes) grown in microfluidic chambers was performed. Single cells were cropped and tracked every 8 min using the ImageJ plug-in MicrobeJ. Phase-contrast, CFP, and YFP signals were recorded separately for analyses of motility and biofilm gene expression of each individual cell during the cultivation. (A) A black cell (no expression of either CFP nor YFP) becomes a motile cell. (B) A black cell that becomes a biofilm former. (C) A black cell that converts to a biofilm former and then to a black cell again. (D) A motile cell which becomes a black cell. (E) A motile cell becoming a biofilm former. (F) A motile cell in which the expression of biofilm genes increases while the expression of motility genes remains constant, i.e., the two genetic programs are expressed simultaneously. The path of selected cells in the microfluidic chamber is shown in video clips in Movie S3. AU, arbitrary units.

The majority (about 60% after 360 min) of wild-type cells expressed the *hag* gene but not *tapA*. A smaller subpopulation (about 9%) expressed *tapA*, whereas neither of the promoters was active in a third subpopulation (Fig. 1). Interestingly, we observed switches in gene expression in all three subpopulations (Fig. 2). Cells initially expressing neither of the two fusions could subsequently go on to activate either the *hag* or the *tapA* promoter (Fig. 2A and B); the expression of *tapA* was activated in some cases, followed by its subsequent inactivation (Fig. 2C). Three distinct switches in behavior were detected for those cells that initially expressed the *hag* gene: the *hag* promoter could be switched off with or without the concomitant induction of the *tapA* promoter (Fig. 2D and E). Finally, the activation of *tapA* in parallel to *hag* was observed in a small proportion (0.3%) of cells, representing a fourth subpopulation (Fig. 2F).

**FIG 3:**
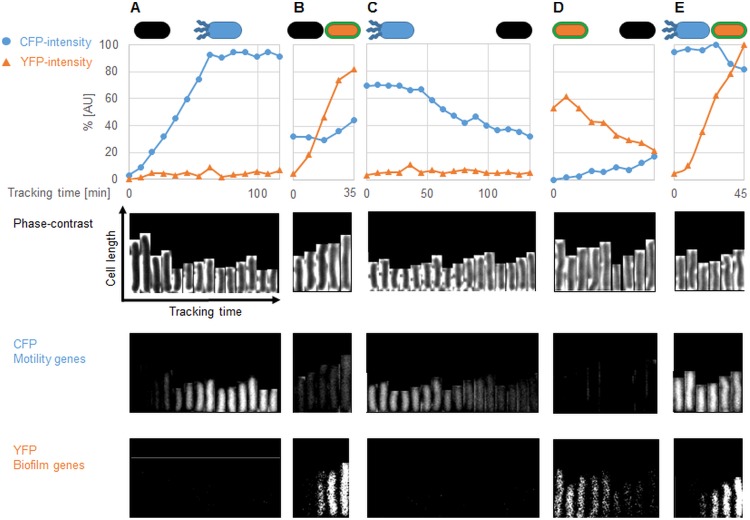
Tracking of single cells during microfluidic single-cell cultivation of B. subtilis *ymdB* mutant. Single-cell tracking of B. subtilis *ymdB* mutant (GP2551) carrying P_*hag*_-*cfp* (motility genes) and P_*tapA*_-*yfp* (biofilm genes) transcriptional fusions grown in microfluidic chambers. Single cells were cropped and tracked every 9 min using the ImageJ plug-in MicrobeJ. Phase contrast, CFP, and YFP signals were separately recorded for analyses of motility and biofilm gene expression of each individual cell during the cultivation. (A) A black cell (no expression, neither CFP nor YFP) becomes a motile cell. (B) A black cell that becomes a biofilm former (suppressor). (C) A motile cell that converts to a black cell. (D) A biofilm former (suppressor) which becomes a black cell. (E) A motile cell in which the expression of biofilm genes increases while the expression of motility genes remains constant as both genetic programs are expressed simultaneously. The path of selected cells in the microfluidic chamber is shown in video clips in Movie S4.

The picture was different for the *ymdB* mutant. Whilst the majority (about 70%, after 360 min) of cells also expressed *hag* (Fig. 1), cells with an active *tapA* promoter appeared only transiently (compare Movie S1 [wild-type cells] and Movie S2 [Δ*ymdB* cells], and Movies S3 and S4 in the supplemental material for the fates of individual cells). Interestingly, the third subpopulation (about 30%) that expressed neither *hag* nor *tapA* in the wild-type strain was similar to that in the *ymdB* mutant, indicating that these cells had entered a gene expression program that was completely independent of YmdB (Fig. 3). Finally, very few cells (0.2%) transiently expressed both genes simultaneously, as observed for the wild type. Taken together, our observations underline the dynamics in a multistable culture in the wild-type strain, which is characterized by the interconversion of the different cell types. Moreover, our findings show that YmdB is required for the expression of biofilm genes in a subpopulation of about 10% of the cells and that this subpopulation also expresses motility genes in the absence of YmdB.

10.1128/mBio.01464-18.6 MOVIE S1  Time-lapse video of immobilized (Δ*hag*) B. subtilis wild-type cells (GP2130) harboring P*_tapA_*-YFP and P*_hag_*-CFP reporter fusions grown in a microfluidic chamber. Cells were grown and recorded in LB medium at 37°C for 512 min. An image was taken every 8 min for phase-contrast signal, CFP signal, and YFP signal. Channels were merged and processed via ImageJ. Download MOVIE S1, AVI file, 11 MB. Copyright © 2018 Kampf et al. 2018 Kampf et al. This content is distributed under the terms of the Creative Commons Attribution 4.0 International license.

10.1128/mBio.01464-18.7 MOVIE S2  Time-lapse video of immobilized (Δ*hag*) B. subtilis *ymdB* deletion mutant (GP2551) harboring P*_tapA_*-YFP and P*_hag_*-CFP reporter fusions grown in a microfluidic chamber. Cells were grown in LB medium at 37°C for 531 min. An image was taken every 9 min for phase-contrast signal, CFP signal, and YFP signal. Channels were merged and processed via ImageJ. Download MOVIE S2, AVI file, 8.7 MB. Copyright © 2018 Kampf et al. 2018 Kampf et al. This content is distributed under the terms of the Creative Commons Attribution 4.0 International license.

10.1128/mBio.01464-18.8 MOVIE S3  Time-lapse videos for different tracked cells of the wild-type strain (GP2130). Cells were tracked using the ImageJ plug-in MicrobeJ and saved as video clips at 1 frame/s. Each frame was taken after a time interval of 8 min. The documented cell is highlighted by a green frame and a pink line, which demonstrates the path of the cell during cultivation. GP2130 contains fusions of P_*hag*_-*cfp* (motility genes) and P_*tapA*_-*yfp* (biofilm genes) for documentation of expression and a *hag* deletion for immobilization. Download MOVIE S3, MPG file, 90.2 MB. Copyright © 2018 Kampf et al. 2018 Kampf et al. This content is distributed under the terms of the Creative Commons Attribution 4.0 International license.

10.1128/mBio.01464-18.9 MOVIE S4  Time-lapse videos for different tracked cells of the *ymdB* mutant (GP2551). Cells were tracked using the ImageJ plug-in MicrobeJ and saved as video clips at 1 frame/s. Each frame was taken after a time interval of 9 min. The documented cell is highlighted by a green frame and a pink line, which demonstrates the path of the cell during growth. GP2551 contains fusions of P*_hag_*-*cfp* (motility genes) and P*_tapA_*-*yfp* (biofilm genes) for documentation of expression and a *hag* deletion for immobilization. Download MOVIE S4, MPG file, 53.1 MB. Copyright © 2018 Kampf et al. 2018 Kampf et al. This content is distributed under the terms of the Creative Commons Attribution 4.0 International license.

### Isolation of *ymdB* suppressor mutants.

During our work with *ymdB* mutants, we noticed the rapid appearance of larger colonies that were able to form wrinkles. We concluded that the *ymdB* mutation triggers the acquisition of suppressor mutations with a restored ability to express biofilm-associated genes. Consequently, suppressor mutants of Δ*ymdB* strains were isolated to understand the molecular mechanism by which YmdB controls heterogeneous gene expression in B. subtilis. The phosphodiesterase activity of YmdB has already been shown to be required for the control of biofilm formation and motility (21), and the suppressor mutants might reveal the molecular target of YmdB.

To isolate suppressors, Δ*ymdB* mutant strains GP846, GP921, and GP1574 (nondomesticated) and GP847 (domesticated) (see Table S1 in the supplemental material for the genotypes of the strains) were passaged several times in LB medium before plating on MSgg medium. The majority of the colonies did not form a matrix; however, a few matrix-forming colonies were observed, indicating that these colonies harbored suppressor mutations that restored biofilm formation in the absence of YmdB.

10.1128/mBio.01464-18.4 TABLE S1  Overview of *ymdB* suppressor mutants. Download TABLE S1, DOCX file, 0.03 MB. Copyright © 2018 Kampf et al. 2018 Kampf et al. This content is distributed under the terms of the Creative Commons Attribution 4.0 International license.

The suppressor mutants were isolated and verified for the *ymdB* deletion. The isolated suppressor mutants were tested for complex colony formation on plates and for pellicle formation in liquid medium. As detailed in Table S1, the GP1561 wild-type strain formed complex colonies as well as robust pellicles, whereas the otherwise isogenic GP1574 *ymdB* mutant was unable to develop these indications of biofilm formation. By contrast, all suppressor mutants tested formed complex colonies and robust pellicles. Analysis of strains carrying reporter gene fusions revealed that the suppressor mutants did indeed express biofilm-associated genes under the control of the *tapA* promoter. These observations indicate that the isolated strains harbored mutations suppressing the phenotype of the Δ*ymdB* mutant. Two of the mutant strains (GP1663 and GP1666; both derived from the domesticated strain GP847) grew in colonies with a mucous appearance, indicating the release of extracellular polysaccharides.

### Identification and phenotypic characterization of the mutations.

**FIG 4:**
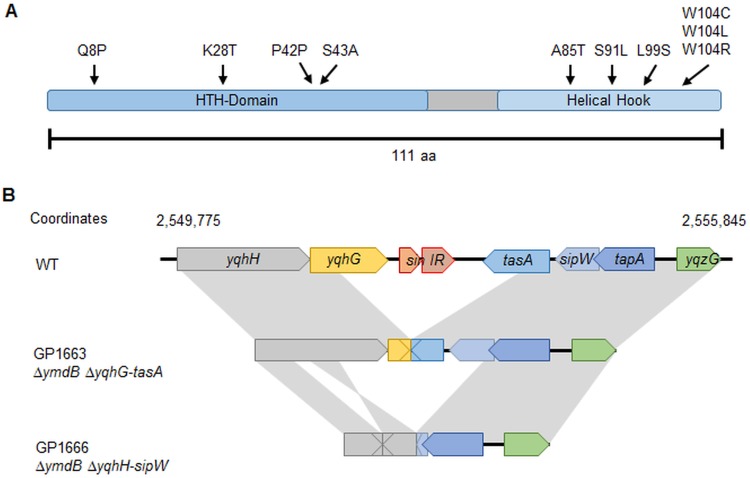
Overview of selected suppressor mutants. (A) The SinR protein with mutations found in the suppressor mutants. (B) Suppressor mutants GP1663 and GP1666 harbored deletions encompassing the *sinIR* operon. Gray areas highlight identical genomic regions in the wild type (GP845) and their relative locations and orientations in the GP1663 and GP1666 suppressor mutants. The GP1663 suppressor mutant showed a 1,760-bp deletion between *yqhG* and *tasA*. GP1666 revealed a large deletion region of about 3500 bp, starting in *yqhH* and ending near the start of *sipW*. Moreover, the suppressor GP1666 harbored another deletion of 22 bp affecting *yqhH* (bp 545 to 566). Another part of the region of *yqhH*, bp 567 to 1319, was reversed.

The *sinR* alleles of all suppressor mutants were amplified and sequenced because it had been shown previously that SinR inactivation restored biofilm formation in a *ymdB* mutant (20). The majority (12 of 14) of the mutants carried point mutations in *sinR* (Table S1). All bar one of the mutations resulted in single amino acid substitutions at different positions throughout the *sinR* coding sequence (Fig. 4A). Five of the 12 *sinR* suppressor mutants affected the tryptophan encoded at position 104, resulting in arginine, cysteine, or leucine substitutions. SinR Trp104-affected suppressor mutants were isolated from *ymdB* mutants of both domesticated and nondomesticated strains. A silent mutation was found in one strain, GP1669, in which the C126T substitution did not affect the encoded amino acid (Pro42). Either this mutation affected the properties of the resulting mRNA, or the strain carried an additional mutation elsewhere in the genome.

**FIG 5:**
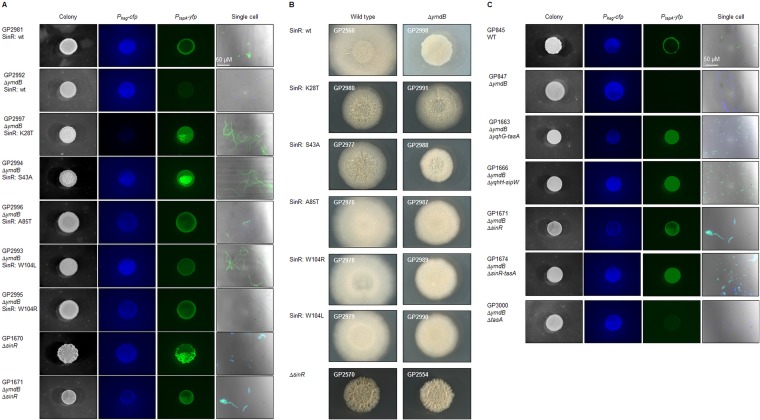
Overview of mutations and deletions found in suppressor mutants leading to restoration of biofilm formation in B. subtilis Δ*ymdB* mutants. Macrocolonies were grown on MSgg agar for 3 days at 30°C. To observe single cells, the bacteria were cultivated in liquid LB medium until an OD_600_ of 1.0 to 1.5 was reached before washing in phosphate-buffered saline (pH 7.5; 50 mM). GP845 and its derivatives contain fusions of P_*hag*_-*cfp* (motility genes) and P_*tapA*_-*yfp* (biofilm genes). (A and B) Macrocolony and single-cell analyses of B. subtilis strains with reintroduced point mutations in SinR found in *ymdB* suppressor mutants (A) into GP845 and its derivatives and (B) into nondomesticated NCIB3610 background and its derivatives (transformable derivative DK1042). (C) Suppressor mutants and constructed strains harboring gene reorganizations. GP1671, GP1674, and GP3000 are strains constructed to investigate the influence of the selected gene deletions on biofilm formation. GP1663 and GP1666 show the effect of deletions and reorganization in the *yqhH* to *yqzG* (encompassing the *sinIR* operon) genomic region on biofilm formation (see Fig. 4B for details). For 1,000-fold magnifications of the single-cell images, see Fig. S2. wt or WT, wild type.

Five representative suppressor mutants with single amino acid substitutions affecting SinR (K28T, S43A, A85T, W104L, and W104R) were analyzed further with respect to colony structure and the expression of motility and biofilm genes at the colony and single-cell levels. The *sinR* mutant alleles were transferred into the background of laboratory strain 168 (carrying fluorescent fusions to the *hag* and *tapA* promoters) and of nondomesticated strain DK1042 to exclude the possibility that the isolated suppressor mutants harbored additional mutations that could affect the outcome of our analysis. All the suppressor mutations restored the expression of the biofilm genes (Fig. 5A) as observed for the *ymdB sinR* double mutant GP1671. All mutations also allowed the formation of complex colonies in the nondomesticated strain background (Fig. 5B). Moreover, the K28T and S43A point mutations in SinR resulted in the activation of both the *hag* and the *tapA* promoters throughout the colonies as seen for the *sinR* deletion mutant. However, while the *sinR* deletion resulted in concomitant expression of both motility (*hag*) and biofilm (*tapA*) genes (Fig. 5A; GP1670), the point mutations affecting Ala85 and Trp104 restored heterogeneous gene expression; i.e., some cells exhibited *hag* promoter activity, whereas others had an active *tapA* promoter, indicating that the corresponding SinR mutant proteins retained some ability to regulate transcription. We assayed the cellular amounts of SinR by Western blotting to confirm the stability of the mutant SinR proteins. The K28T and S43A mutant proteins were present at levels similar to the wild-type levels in isogenic backgrounds, whereas A85T, W104L, and W104R protein levels were a little higher than the wild-type levels (see Fig. S3 in the supplemental material). The L99S variant could not be identified in Western blotting (data not shown), indicating that this protein was unstable, and it was not studied further.

The *sinR* gene could not be amplified for mucous mutants GP1663 and GP1666. Amplicons were obtained for them that were substantially smaller than those obtained for wild-type chromosomal DNA using primers specific for the chromosomal locations on either side of the *sinR* open reading frame. An ~1.8-kbp region was deleted in GP1663 that encompassed *yqhG* to *tasA*, the genes upstream and downstream of *sinR*, respectively. The deleted region was even larger in GP1666, approximately 3.5 kbp, extending from *yqhH* to *sipW*. One part of *yqhH*, which is retained in the *ymdB* mutant, had been inverted in strain GP1666 in comparison to the wild-type strain (Fig. 4B). The loss of *tasA*, in addition to the *sinR* deletion, could be responsible for the mucous colony appearance because TasA forms amyloid fibers in the extracellular biofilm matrix. To test this hypothesis, the *sinR*, the *sinR-tasA*, and the *tasA* chromosomal regions were replaced with an antibiotic resistance gene and the phenotypes of the resulting strains, GP1671, GP1674, and GP3000, respectively, were assessed. *ymdB sinR* double mutant GP1671 formed complex colonies (Fig. 5C), although the effect was less pronounced than that seen with wild-type strain GP845. The *ymdB sinR* double mutant expressed the motility and biofilm genes simultaneously, confirming prior results. The deletion of *tasA* alone in the *ymdB* background was insufficient to restore the mucous phenotype. Only the additional deletion of *sinR* (see GP1674 results) resulted in the formation of very mucous colonies, as observed for the two suppressor mutants, indicating that the simultaneous absence of *tasA* and *sinR*, to permit expression of the extrapolysaccharide matrix, was responsible for the phenotype.

### Biochemical characterization of the SinR variants.

The phenotypic analysis of the SinR variants revealed that they had retained some activity. The corresponding alleles were cloned, and the recombinant proteins were purified and analyzed with respect to DNA binding, oligomerization, and interaction with the SinR antagonist, SinI.

**FIG 6:**
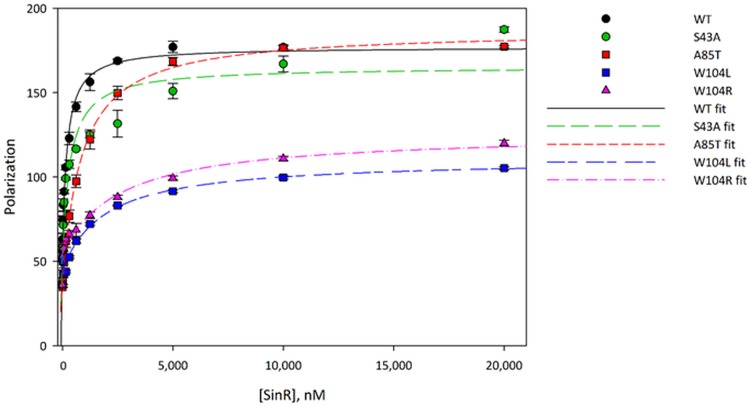
Binding of wild type and suppressor SinR variants to DNA. Fluorescence polarization was used to determine the dissociation constants (*K*_*D*_) of SinR variants binding to operator DNA. Fluorescently labeled DNA (10 nM concentration) was incubated with various concentrations of SinR proteins, and the fluorescence polarization was measured in triplicate. The polarization data were fitted to a 1:1 binding model in Sigma Plot software to determine *K*_*D*_ values, which are reported in Table 1.

DNA binding was measured by fluorescence polarization using a fluorescein-labeled 21-bp DNA duplex containing a pair of inverted repeats of the consensus SinR binding sequence, the *sin* box (GTTCTCT), from the *eps* promoter. Fitting the polarization data with a 1:1 binding model yielded a dissociation constant of 180 nM for the interaction with the wild-type protein, in reasonable agreement with the value of 360 nM previously measured by isothermal titration calorimetry (13). Dissociation constants for DNA binding were determined for all of the SinR variants (Fig. 6; see also Table 1). Notably, DNA binding of the SinR^W104L^ and SinR^W104R^ mutants was reduced 10-fold, whereas the SinR^S43A^ and SinR^A85T^ mutations reduced DNA binding affinity 2- and 5-fold, respectively. No significant change in polarization was observable for SinR^K28T^, even at protein concentrations of up to 20 µM, indicating a complete loss of DNA-binding activity for this particular variant (data not shown).

**TABLE 1:** Overview of DNA binding and oligomerization of SinR variants

SinR variant	Binding of DNA motif (*K_D_*^a^ [nM]) by fluorescence polarization	Oligomerization status by SEC-MALS
Wild type	179 ± 24	Tetramer
K28T	No binding	Tetramer
S43A	350 ± 81	Tetramer
A85T	852 ± 39	Dissociating/unstable tetramer
W104L	1,896 ± 298	Dimer
W104R	2,580 ± 545	Dimer

^a^ *K*_*D*_, dissociation constants.

**FIG 7:**
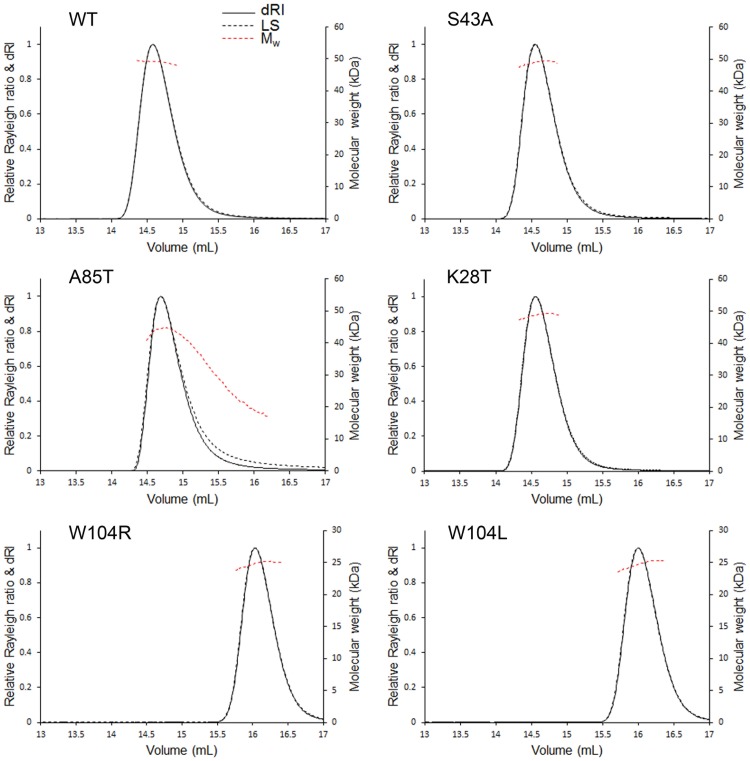
Oligomerization analysis of SinR variants via SEC-MALS. Absolute molar masses of proteins were determined through size exclusion chromatography multiangle static light scattering (SEC-MALS). The purified SinR proteins were loaded onto a Superdex 200 Increase 10/300 size exclusion chromatography column for SEC-MALS analysis. Data were collected and analyzed using ASTRA 6 software (Wyatt Technology). Molecular masses were calculated across eluted protein peaks through extrapolation from Zimm plots using a dn/dc value of 0.1850 ml/g; quoted molecular weights and estimated errors relate to the overall mass calculation across a single peak.

The oligomeric status of the mutated SinR proteins was analyzed by size exclusion chromatography with multiangle static light scattering (SEC-MALS) using proteins at a concentration of 5 mg/ml, equivalent to 400 µM (Fig. 7). SEC-MALS chromatograms of wild-type SinR, SinR^K28T^, and SinR^S43A^ all showed a single symmetrical peak corresponding, from the uniform deconvoluted molecular weight of ~50 kDa across the peak, to a tetramer (SinR monomers are 12.85 kDa). The central peak on the SEC-MALS chromatogram was less symmetrical for SinR^A85T^, containing species with molecular weights ranging between ~45 and ~20 kDa, suggesting that the tetramer formed by SinR^A85T^ was less stable than that seen with the wild-type strain and had dissociated during the size exclusion chromatography process. The chromatograms for the SinR^W104L^ and SinR^W104R^ mutants had a single symmetrical peak from which a deconvoluted molecular weight of 25 kDa was obtained, corresponding to a dimer.

**FIG 8:**
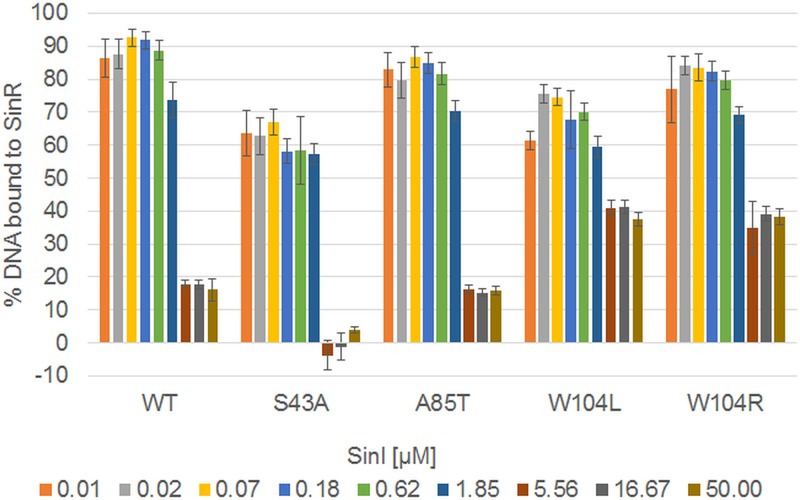
Inhibition of SinR variants at different SinI concentrations. SinI was titrated against SinR variants prebound to the native operator site in order to analyze the ability of SinR proteins to bind to the antagonist SinI. Fluorescently labeled DNA (10 nM concentration) was preincubated with 5 µM of SinR, followed by a SinI titration and the measurement of fluorescence polarization. The polarization data at various SinI concentrations have been normalized to represent the percentages of DNA bound to SinR based on the measured levels of polarization of both free DNA and the DNA:SinR complex; for all of the SinR proteins, the DNA binding is saturated at 5 µM SinR and 10 nM DNA. The bars show the mean values and standard deviations of three measurements.

The interaction of the SinR mutants with SinI was assessed qualitatively by measuring the displacement of SinR-bound DNA by SinI using fluorescence polarization (Fig. 8). Nearly complete displacement of the DNA was obtained upon adding an equimolar amount of SinI to the SinR:DNA complex; ~85% of the bound DNA was released. The incomplete displacement of DNA, despite the 30-fold-higher affinity of SinR for SinI than for its inverted repeat DNA target (13), probably reflects the slow kinetics of the SinR:SinI association (13) even though the fluorescence polarization experimental setup was conducted on a 10^2^–10^3^-s time scale. The incomplete displacement of the DNA observed here is consistent with similar results in competitive surface plasmon resonance experiments reported previously (12). The SinR^A85T^ protein behaved similarly to results seen with the wild-type strain, while displacement of the DNA from its complex with SinR^S43A^ was almost complete when SinI was present at levels that were equimolar with respect to or higher than those of SinR^S43A^. The displacement of the DNA was inefficient for the SinR^W104L^ and SinR^W104R^ variants. Only about 60% of the bound DNA was released even in the presence of a 10-fold molar excess of SinI over SinR, implying that the W104 mutations slowed the kinetics of the SinR:SinI interaction whereas that of S43A increased the association rate. The SinI-induced displacement of SinR from DNA observed here is unlikely to have been a consequence of SinI binding directly to DNA; in this scenario, the W104 SinR variants would be displaced more easily from DNA because they bind it 10-fold more weakly than the wild type (Fig. 6; see also Table 1), but we observed the opposite phenomenon. Therefore, it follows that the displacement of SinR from DNA occurred because SinI binds to SinR to disrupt its multimerization. While SinR is a tetramer in solution, the SinI:SinR complex is a heterodimer (12, 16); therefore, SinI binding necessitates dissociation of the subunits in the SinR tetramer.

## DISCUSSION

The YmdB phosphodiesterase is required for biofilm formation in B. subtilis. In a *ymdB* mutant, most cells express the genes necessary for motility and chemotaxis but not those for biofilm formation (20, 21) (Fig. 1). As the formation of the extracellular polysaccharide and protein matrix is certainly not required for life under laboratory conditions, and since biofilm formation is a trait of B. subtilis lost during domestication (26, 27), it is tempting to speculate that *ymdB* mutants may have a selective advantage in the artificial setting of the laboratory. However, *ymdB* mutant cells of B. subtilis readily acquired suppressor mutations that partially or fully restored biofilm formation, which suggests that the cells undergo selective pressure to restore biofilm gene expression and matrix production. Note that this selective pressure occurred under laboratory conditions with both domesticated and nondomesticated strains. It has already been suggested that the acquisition of mutations that facilitate biofilm formation may provide a fitness benefit for B. subtilis (28). Alternatively, the selective pressure might be caused by the lack of a SinR-repressed gene, and biofilm formation may be a by-product resulting from the restoration of this gene by its mutation. This hypothesis, however, is rather unlikely; in saturating suppressor screens that involve regulatory events, mutations typically affect both the transcription factor and its target site (4, 6, 29). As SinR represses two distinct and unlinked operons required for biofilm formation, the *eps* and the *tasA* operons for extracellular polysaccharide synthesis and for production and export of the amyloid protein TasA, respectively, mutations affecting the SinR binding site of only one operon would be insufficient to restore biofilm formation. The exclusive occurrence of mutations affecting SinR in all analyzed mutants indicates that the selective pressure is directed toward expression of multiple and independent SinR target operons and that biofilm formation is the relevant function.

The inspiration of this study was that the suppressor mutations would help to identify the molecular target of YmdB, as the mutations might alter the target in such a way to restore complex colony development and biofilm formation. If the YmdB function was the phosphodiesterase activity-mediated degradation of a second messenger nucleotide, suppressor mutants that prevented the synthesis of the corresponding nucleotide could accumulate. However, all the suppressors affected the expression or activity of SinR, indicating that the homeostasis of SinR or its antagonist proteins SinI and SlrR is the major function of YmdB.

A molecular explanation of the effects of the mutations in SinR can be provided by reference to the biochemical results and to the crystal structures of the SinR:SinI complex (16), the isolated N- and C-terminal domains of SinR (12), and the SinR:DNA complex (13). There are two contrasting proposals for SinR tetramer formation (12, 13), and the data presented in this study shed new light on which has physiological relevance.

Our *in vivo* studies demonstrated that the replacements at positions 28 and 43 result in inactive proteins, an observation supported by the biochemical data. SinR^K28T^ has no DNA binding capacity, consistent with the role of SinR^K28^ in *sin* box recognition (see Fig. S1A in the supplemental material) (13). SinR^S43A^ has reduced DNA-binding affinity and can be more easily displaced from DNA by SinI than wild-type SinR, presumably because the dimer interface of SinR bound to inverted *sin* box pairs (13) is affected by this mutation (Fig. S1B).

10.1128/mBio.01464-18.1 FIG S1  Structural analysis of the effects of suppressor mutations in SinR. (A) The Lys28Thr mutation has a marked effect on DNA binding because of the loss of the contact between SinR and the O6 atom of the first base, G1, in the *sin* box. Note that in the structure of the SinR:DNA complex (PDB identifier [ID] 3ZKC), the *sin* box G1 base corresponds to G4 in the sequence of the cocrystallized oligonucleotide. Both DNA (green) and protein (cyan) are drawn in cartoon fashion, and atoms are colored red for oxygen and dark blue for nitrogen; carbons are green in the DNA and cyan in the protein. (B) The Ser43Ala mutation has a slight negative impact on DNA binding affinity because of the loss of the contact to the phosphate of the *sin* box G6 base (equivalent to G14 in the sequence of the cocrystallized oligonucleotide) and the likely impact on the protein dimer interface; the DNA is colored by atom as described for panel A, and the two protein chains are colored cyan and green. (C) The hydrophobic environment surrounding Ala85 is illustrated by depicting its neighbors—Trp78, Phe95, and Leu99—in stick format. Each chain of the C-terminal domain of SinR in the tetramer (as described by Colledge et al. [12]) (PDB ID 2YAL) is colored independently. Download FIG S1, PPT file, 1.5 MB. Copyright © 2018 Kampf et al. 2018 Kampf et al. This content is distributed under the terms of the Creative Commons Attribution 4.0 International license.

The SinR^A85T^ protein formed an unstable tetramer in solution (Fig. 7), which is explained by the structure of the isolated C-terminal domain of SinR (12). The side chain of Ala85 from one protomer in a SinR dimer is found in a hydrophobic environment involving Trp78, Phe95, and Leu99 (which was mutated to serine in one of the other suppressor mutants, resulting in an unstable protein) from the other protomer (Fig. S1C). The introduction of the bulkier threonine probably leads to a reorganization of the hydrophobic core around residue 85. Phe95 packs against Phe98 at the dimer interface; therefore, both the dimer:dimer and monomer:monomer interfaces in the SinR tetramer are destabilized in SinR^A85T^, consistent with the SEC-MALS analysis (Fig. 7). The unstable SinR^A85T^ tetramer probably explains its 4-fold-reduced affinity for DNA containing an inverted *sin* box pair, given that wild-type SinR binds this sequence as a tetramer (12). Note that the strains expressing SinR^A85T^ exhibit bistability of motility and biofilm gene expression (Fig. 5A), which may result from the reduced, but not completely lost, SinR repression activity *in vivo*.

**FIG 9:**
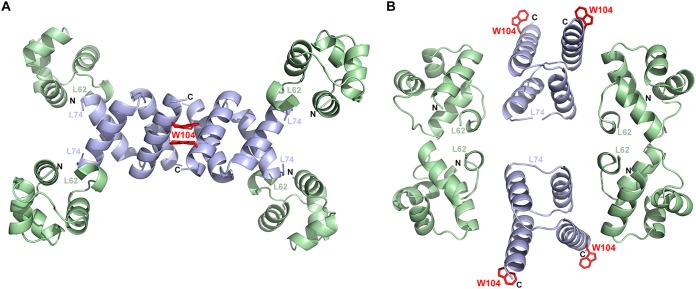
W104 mutants discriminate between different SinR tetramer models. Ribbon representations are shown of the two proposed models of SinR tetramers, in which the DNA binding domains are colored pale green and the tetramerization domains are colored pale blue. Dashed lines represent the linkers between the domains that cannot be modeled in any SinR-containing structure because of flexibility. The N and C termini are labeled, where they are visible, and the side chain for W104 is drawn in “stick” format and colored and labeled in red. For the model in panel A (from Colledge et al. [12] PDB ID 2YAL), the structure of the C-terminal domain of SinR was solved in isolation. Note that the DNA-binding domains of SinR in this model are too far apart to be consistent with binding to pairs of *sin* boxes as found in promoters of genes regulated by SinR, but the position of W104 in this model, critical to tetramerization, is consistent with the biochemistry and genetics presented here. For the model in panel B (from Newman et al. [13] PDB ID 3ZKC), the structure of SinR bound to DNA is described; for clarity, the DNA is not included in this panel. Note that in the model in panel B, W104 plays no role in self-assembly of SinR.

Finally, several mutations affecting SinR^W104L^ restored the biofilm phenotype to the *ymdB* deletion in parental domesticated and nondomesticated strains, including substitutions by arginine and leucine. Both these SinR variants could form only dimers (Fig. 7). Two contrasting suggestions have been made regarding the tetramerization of SinR. The first proposes that the four C-terminal helices in the structure of the isolated tetrameric C-terminal domain of SinR (12) associate loosely as two semiorthogonal pairs of antiparallel helices in which pairs of Trp104 side chains stack against one another and Tyr101 to stabilize the tetramer (Fig. 9A). In the second, an alternative tetramer has been proposed (13) based upon residual electron density for the disordered C-terminal helices of SinR in the SinR:DNA complex, but Trp104 is not involved in any interface in this model (Fig. 9B). Trp104 plays a critical role in SinR tetramerization (Fig. 7), and because of the loss of important self-contacts and contacts to Tyr101, the Trp104 variants do not form tetramers. Therefore, the SinR tetramerization model of Colledge et al. (12) is likely correct. The 10-fold reduction in DNA binding of the SinR^W104^ variants in comparison to wild-type SinR (Fig. 6) is consistent with these variants binding to DNA as dimers instead of as wild-type tetramers. This finding is also in agreement with the observed bistable gene expression of motility and biofilm genes that indicates that these proteins retain some repression activity *in vivo*. Finally, SinI was less effective at displacing SinR^W104^ variants from DNA than wild-type SinR (Fig. 8), indicating that Trp104, though not involved directly in the formation of a stable complex with SinI (16), must be involved in an intermediary step when SinR multimers dissociate to form the thermodynamically dead-end SinI:SinR complex.

The results presented in this study suggest that YmdB rather directly affects the homeostasis of SinR and/or its antagonist proteins. How could such control be exerted? As YmdB is a phosphodiesterase and as this activity is essential for bistable gene expression, this enzyme ought to cleave a substrate containing phosphodiesters such as second messenger nucleotides, glycerophosphodiesters found in lipids, or nucleic acids. Our previous work has excluded the possibility of the involvement of YmdB-hydrolysable second messengers in the control of biofilm formation (21), and no link between glycerolphosphate and SinR activity has been detected to date. YmdB may thus control the SinR switch by acting directly on nucleic acids. Indeed, the *sinI-sinR* and *sinR* transcripts are controlled at the posttranscriptional level by their degradation by RNase Y-containing protein complexes (30–32). Elucidating the links between YmdB and *sinI-sinR* mRNA stability will be the subject of our future work.

## MATERIALS AND METHODS

### Bacterial strains and growth conditions.

The B. subtilis strains were derived from laboratory strain 168 (*trpC2*) or from nondomesticated wild-type strain NCIB3610. All strains are listed in Table S2A in the supplemental material. Escherichia coli XL1-Blue (Stratagene) and BL21(DE3) (33) were used for cloning experiments and protein overproduction, respectively. E. coli was routinely grown in lysogeny broth (LB) (33) at 37°C. B. subtilis was grown in SP (sporulation) medium or in LB medium (34). LB plates were prepared by addition of 17 g Bacto agar/liter (Difco) to LB (33). When required, media were supplemented with the following antibiotics (concentrations): ampicillin (100 µg/ml) or kanamycin (50 µg/ml) (for E. coli); spectinomycin (150 µg/ml), kanamycin (10 µg/ml), tetracycline (12.5 µg/ml), chloramphenicol (5 µg/ml), and erythromycin (2 µg/ml) plus lincomycin (25 µg/ml) (for B. subtilis).

10.1128/mBio.01464-18.5 TABLE S2  Bacterial strains (A), oligonucleotides (B), and plasmids (C) used in this study. Download TABLE S2, DOCX file, 0.04 MB. Copyright © 2018 Kampf et al. 2018 Kampf et al. This content is distributed under the terms of the Creative Commons Attribution 4.0 International license.

### DNA manipulation and transformation.

Transformation of E. coli and plasmid DNA extraction were performed using standard procedures (33). Restriction enzymes, T4 DNA ligase, and DNA polymerases were used as recommended by the manufacturers. DNA fragments were purified by using a QIAquick PCR purification kit (Qiagen, Germany). Phusion DNA polymerase was used for the PCR as recommended by the manufacturer. All primer sequences are provided as supplemental material (Table S2B). DNA sequences were determined using the dideoxy chain termination method (33). All plasmid insertions derived from PCR products were verified by DNA sequencing. Chromosomal DNA of B. subtilis was isolated using a peqGOLD bacterial DNA kit (Peqlab, Erlangen, Germany). Standard procedures were used to transform E. coli (33), and transformants were selected on LB plates containing ampicillin (100 µg/ml) or kanamycin (50 µg/ml). B. subtilis was transformed with chromosomal DNA or PCR products according to the two-step protocol (35). To transfer mutations into the background of nondomesticated wild-type strain NCIB3610, SPP1-mediated phage transduction was performed as described previously (20). Transformants and transductants were selected on SP plates containing the appropriate antibiotics.

### Construction of deletion strains.

Deletion of the *hag* and *tasA* genes was achieved by transformation with PCR products constructed using oligonucleotides (Table S2B) to amplify DNA fragments flanking the *hag* and *tasA* genes and intervening tetracycline and chloramphenicol resistance cassettes as described previously (36, 37).

### Transfer of *sinR* point mutations to wild-type strains.

To construct a set of isogenic strains carrying the *sinR* wild-type and mutant alleles, PCR products of the upstream *sinR* region (from the wild-type and mutant strains), a tetracycline resistance determinant from plasmid pDG1514, and the downstream *tasA* region were used to transform strains GP845 and DK1042 and their isogenic *ymdB* mutant derivatives as described previously (36, 37). The resulting strains are listed in Table S2A.

### Plasmid constructions.

The SinR variant proteins were expressed in E. coli BL21(DE3) for subsequent assessment of their biochemical properties. The *sinR* alleles were amplified from chromosomal DNA of the respective mutants using primer pair G8/G10 (13) and were cloned between the NdeI and EcoRI sites of expression vector pET24a (Novagen). The resulting plasmids and the corresponding mutations are listed in Table S2C. The *sinI* gene was amplified by PCR using primer pair G6/G7 and cloned into plasmid pC2 (13).

### Protein purification.

The SinR variants were overexpressed in E. coli BL21(DE3) transformed with the corresponding expression plasmids. The cultures were grown in flasks of 1 liter LB medium at 37°C. Expression was induced by the addition of IPTG (isopropyl-β-d-thiogalactopyranoside) (final concentration, 1 mM) to logarithmically growing cultures (optical density at 600 nm [OD_600_] of 0.5), and cultivation was continued for 1 h. Cells were harvested, and the pellets from 2 liters of culture medium were resuspended in 20 ml disruption buffer (50 mM Tris-HCl, pH 8.0). The cells were lysed by the use of sonication or a OneShot cell lysis kit before insoluble cellular debris was pelleted by centrifugation. The supernatant was filtered through a 0.45-µM pore size syringe filter before loading onto a heparin Sepharose (GE Healthcare) pseudoaffinity column, preequilibrated in 50 mM Tris-HCl (pH 8.0). The bound proteins were eluted using a linear NaCl gradient, from 0 to 1 M NaCl, over 20 column volumes. Those fractions that were determined by SDS-PAGE to contain SinR proteins were pooled, concentrated, and further purified by size exclusion using a Superdex 75 HR 16/60 (GE Healthcare) gel filtration column, preequilibrated in 50 mM Tris-HCl (pH 8.0), 250 mM NaCl. The SinR-containing fractions were pooled, concentrated, and snap-frozen in small aliquots in liquid nitrogen for storage at −80°C. SinI was expressed, resuspended, lysed, and clarified as described above. The clarified cell lysate was loaded onto an ANX (GE Healthcare) ion exchange column and purified by the application of a linear, 0 to 1 M NaCl gradient. Those fractions that were determined by SDS-PAGE to contain SinI proteins were pooled, concentrated, and further purified by size exclusion as described above. The SinI-containing fractions were pooled, concentrated, and snap-frozen in small aliquots in liquid nitrogen for storage at −80°C.

### Determination of protein molecular mass.

The purified proteins were concentrated to 5 mg/ml for SEC-MALS analysis of their absolute molecular masses. Samples (150 μl) of each SinR protein were loaded onto a Superdex 200 Increase 10/300 GL size exclusion chromatography column (GE Healthcare) preequilibrated in 50 mM Tris-HCl (pH 8.0), 250 mM NaCl, attached to an Äkta Pure chromatography workstation (GE Healthcare). The chromatogram was developed at a flow rate of 0.5 ml/min, and the eluent was fed directly into a Dawn Heleos II MALS detector (Wyatt Technology), operating with a laser source of 664 nm and 8 fixed-angle detectors. Absolute and differential refractive indices (dRI) were also measured at 664 nm at 25°C using an Optilab T-rEX differential refractometer (Wyatt Technology). Data were collected and analyzed using ASTRA 6 software (Wyatt Technology).

### Fluorescence polarization.

Oligodeoxynucleotides (FAM1721, labelled at the 5' terminus with fluorescein, and C1723) (50 μM) were annealed with a concentration that was an equimolar equivalent of that of their unlabeled complements in a buffer of 20 mM Tris-HCl (pH 8.0), 100 mM NaCl, 1 mM EDTA by heating the mixture to 95°C for 10 min, followed by slow cooling to room temperature for at least 30 min. For fluorescence polarization, a 10 nM concentration of labeled DNA duplex was mixed with 20 µM SinR in a buffer of 10 mM Tris-HCl (pH 8.0), 100 mM NaCl, 1 mM EDTA and subsequently serially diluted with a 10 nM concentration of labeled DNA duplex in the same buffer. Fluorescence polarization was measured in a PHERAstar FS plate reader using Corning 384-well low-volume black round-bottom polystyrene New Brunswick (NB) microplates. The fluorescence polarization data were fitted to a 1:1 binding model to calculate an equilibrium dissociation constant using SigmaPlot (Systat Software, Inc.). For the SinI titration against SinR-bound DNA, a mixture of 50 µM SinI, 5 µM SinR, and 10 nM DNA was serially diluted against a solution of 5 µM SinR and 10 nM DNA to titrate the SinI concentration from 50 µM to 10 nM.

### Western blotting.

Proteins were separated by 12% SDS-PAGE and transferred onto polyvinylidene difluoride (PVDF) membranes (Bio-Rad) by electroblotting for Western blot analysis. Rabbit anti-SinR (11) served as primary antibodies. The antibodies were visualized by using anti-rabbit immunoglobulin alkaline phosphatase secondary antibodies (Promega) and a CDP-Star detection system (Roche Diagnostics), as described previously (38).

### Assays of complex colony formation.

For colony architecture analysis, bacteria were precultured in LB until an OD_600_ of 0.6 to 0.8 was achieved. The culture (1.5 ml) was then pelleted and resuspended in 100 µl of the sterile supernatant. A 5-µl volume of this cell suspension was spotted onto minimal pellicle MSgg medium as described by Branda et al. (39) containing 1.5% agar and incubated at 30°C for 3 days.

### Microscopy.

For fluorescence microscopy, cells were grown in LB medium to an OD_600_ of 0.7 to 1.0, harvested, and resuspended in phosphate-buffered saline (pH 7.5; 50 mM). Fluorescence images were obtained with an Axioskop 40 FL fluorescence microscope equipped with an AxioCam MRm digital camera and AxioVision Rel 4.8 software for image processing (Carl Zeiss, Inc., Göttingen, Germany) and a Neofluar series objective at ×100 primary magnification. The applied filter sets were a YFP HC filter set (AHF Analysentechnik, Tübingen, Germany) (BP [band pass], 500/24; FT [dichroic beam-splitting mirror], 520; LP [long pass], 542/27) for YFP detection and filter set 47 (Carl Zeiss, Inc.) (BP, 436/20; FT 455; LP, 480/40) for CFP visualization. All images were taken using the same exposure time. The overlays of fluorescent and phase-contrast images were prepared for presentation with Adobe Photoshop Elements 8.0 (Adobe Systems, San Jose, USA).

To monitor gene expression in complex colonies, plates were incubated in the dark, and biofilm assay results were documented with a digital reflex camera (Olympus) and a stereo microscope (Carl Zeiss, Inc.) equipped with an AxioCam MRc digital camera. Micrographs were taken at 9.6-fold magnification and processed with ZEN 2012 (blue edition) software (Carl Zeiss, Inc.). Photographs were taken using an exposure time of 2 s with Lumar filter set 47 CFP (E) (BP, 436/25; BP, 480/40) or with an exposure time of 1 s and Lumar filter set 46 YFP (E) (BP, 500/20; BP, 535/30).

### Microfluidic cultivation and analysis.

The microfluidic device used in this study was designed for continuous microcolony growth and phenotypic studies at the single-cell level. Master molds and disposable polydimethylsiloxane (PDMS)-glass chips were produced and microfluidic devices assembled as previously described (40). For this study, the growth chamber design (41) was modified to meet the requirements for cultivating B. subtilis. The system contained 400 chambers in parallel arrays (8 by 50) with a chamber size of 80 µm by 90 µm (with two sides open to the main channel). Microfluidic single-cell cultivation and analysis were performed as previously described (40, 42). For microfluidic cultivations, cells were first grown in an overnight LB culture and transferred to a main culture. There, cells were grown to an OD_600_ of 0.4 to 0.6 and inoculated into the chip as previously described (43). Chambers were manually selected for time-lapse imaging, and the growth at 37°C was imaged for 24 h with a continuous supply of LB medium (300 nl/min). Fluorescence images were obtained with a Nikon Ti-E Eclipse fluorescence microscope (Nikon, Japan). Fluorescence and phase-contrast images were taken using an Andor Luca R charge-coupled-device (CCD) camera (Andor Technology Ltd., Northern Ireland) in combination with a 100× objective (Nikon, Japan) (Plan Apochromat λ oil; numerical aperture [NA] = 1.45; working distance [WD] = 170 µm). An Intensilight mercury-vapor lamp (Nikon, Japan) was used as the fluorescence excitation (EX) light source. Phase contrast, YFP, and CFP images were taken every 8 or 9 min using YFPHQ filters (EX, 490 to 550 nm; dichroic mirror [DM], 510 nm; barrier filter [BA], 520 to 560 nm) and HQCFP filters (EX, 420 to 445 nm; DM, 450 nm; BA, 460 to 510 nm). The exposure times were 50 ms for the phase-contrast images and 200 ms for the fluorescence images. The mean fluorescence values for each cell (see Fig. 1 and 2) were analyzed using the ImageJ plug-in MicrobeJ (44).

10.1128/mBio.01464-18.2 FIG S2  Magnified (1,000-fold) images of single-cell analysis. (Left panel) B. subtilis strains harboring reintroduced point mutations in SinR as found in *ymdB* suppressor mutants. (Right panel) Suppressor mutants and constructed strains harboring gene reorganizations of the *sinIR* genomic region. All strains contained fusions of P_*hag*_-*cfp* (motility genes) and P_*tapA*_-*yfp* (biofilm genes). Download FIG S2, PPT file, 2.1 MB. Copyright © 2018 Kampf et al. 2018 Kampf et al. This content is distributed under the terms of the Creative Commons Attribution 4.0 International license.

10.1128/mBio.01464-18.3 FIG S3  SinR protein stability in the wild-type strains, *ymdB* mutants, and evolved suppressor mutants. Western blotting for determination of SinR amounts in *ymdB* suppressors with antibodies against SinR as the protein of interest and antibodies against GapA as a loading control. SinR amounts were documented in wild-type strains, isogenic *ymdB* mutants, and evolved suppressor mutants in comparison. Download FIG S3, PPT file, 0.6 MB. Copyright © 2018 Kampf et al. 2018 Kampf et al. This content is distributed under the terms of the Creative Commons Attribution 4.0 International license.
