# Supplementary material for: Selective Pressure for Biofilm Formation in Bacillus subtilis: Differential Effect of Mutations in the Master Regulator SinR on Bistability
Source: mBio. 2018 Sep 4;9(5):e01464-18. doi: 10.1128/mBio.01464-18 (PMC6123443; doi:10.1128/mBio.01464-18)
Supplement: TABLE S1 [file mbo004184042st1.docx]

**Table S1. Overview of *ymdB* suppressor mutants**

Reporter systems: P_tapA_-yfp = matrix gene reporter; P_hag_-cfp = motility gene reporter

| **Strain** | **Genotype / Mutation** | **Biofilm formation** | **Pellicle formation** | **Expression of biofilm and motility genes (colony level)** | **Fluorescence**  **microscopy**  **(single cell level)** |
| --- | --- | --- | --- | --- | --- |
| **DL382**  **(168, wild type)** | P_tapA_-yfp | Structured colony with a rough surface and wrinkles. | Robust structured pellicle. | Expression of matrix genes of the whole colony. | Small cells with and without chaining. Some chaining cells showed expression of matrix genes. |
| **GP846** | ∆*ymdB*  P_tapA_-yfp | Smooth, shiny and unstructured colony. | No pellicle. | No expression of matrix genes. | No expression of matrix genes. |
| **GP1667** | ∆*ymdB*  P_tapA_-yfp  SinR: W104C | Structured colony with a rough surface, without wrinkles. | Structured pellicle, but weaker than the wild type. | Strong matrix genes expression at the outer area of the colony; weak expression in the center. | Mostly long cells and cell chains. Small subpopulation of small cells expressed matrix genes. |
| **GP1668** | ∆*ymdB*  P_tapA_-yfp  SinR: S91L | Vaguely resembled the wild type biofilm structure. Strongly structured center of the colony. | Thin pellicle with small cell aggregates as floating flakes. | Matrix genes expression as described for the wild type strain. | Expression of matrix genes in half of the cell population. Cells were elongated and formed extensive chains. |
| **GP1669** | ∆*ymdB*  P_tapA_-yfp  SinR: P42P | Weak matrix expression, unstructured colony, without wrinkles. | Robust structured pellicle as for the wild type. | Strong matrix genes expression at the inner region of the colony, weak expression in the outer region. | Expression of matrix genes in about 60 % of the cells. Cell were small and showed no chaining. |
| **GP845**  **(168, wild type)** | P_hag_-cfp  P_tapA_-yfp | Structured colony with a rough surface and wrinkles. | Robust structured pellicle. | P_hag_-cfp expression of the inner region of the colony. P*_tapA_*-*yfp* expression of the whole colony. | Most cells showed motility genes expression or no expression of reporter genes. A small subpopulation of cells showed matrix genes expression. Bistable expression. |
| **GP847** | ∆*ymdB*  P_hag_-cfp  P_tapA_-yfp | Smooth, shiny and unstructured colony. | No pellicle. | P_hag_-cfp expression of the whole colony. | Most cells showed motility genes expression. Several cells showed no expression. No matrix gene expression. |
| **GP1663** | ∆*ymdB*  P_hag_-cfp  P_tapA_-yfp  ∆*yqhG-tasA* | Small and mucous colony. | Delicate pellicle. | Matrix and motility genes expression of the whole colony. | Small cells with matrix and motility genes expression. No bistable expression pattern. |
| **GP1664** | ∆*ymdB*  P_hag_-cfp  P_tapA_-yfp  SinR: Gln8Pro | Small, rough and structured colony. | Robust and very structured pellicle. | Strong and matrix genes expression of the whole colony. Weak motility genes expression. | Long cells with matrix and motility genes expression. |
| **GP1665** | ∆*ymdB*  P_hag_-cfp  P_tapA_-yfp  SinR: W104L | Small, rough colony without wrinkles. | Broken and unstructured pellicle. | Matrix and motility genes expression of the whole colony, but weaker than the wild type. | Most of the cells split in motility genes expressing and no reporter gene expressing cells. A small subpopulation of cells showed matrix genes expression. Bistable expression. |
| **GP1666** | ∆*ymdB*  P_hag_-cfp  P_tapA_-yfp  ∆*yqhH-sipW* | Small and mucous colony. | Delicate pellicle. | Matrix and motility genes expression of the whole colony. | Small cells with matrix and motility genes expression. No bistable expression pattern. |
| **NCIB3610**  **(wild type)** |  | Structured colony with a rough surface and wrinkles. | Robust structured pellicle. | - | - |
| **GP921** | ∆*ymdB* | Smooth, shiny and unstructured colony. | No pellicle. | - | - |
| **GP1657** | ∆*ymdB*  SinR: S43A | Matrix producing colony; less pronounced wrinkles than the wild type. | Robust structured pellicle. | - | - |
| **GP1658** | ∆*ymdB*  SinR: W104R | Structured colony without wrinkles. | Weak structured pellicle. | - | - |
| **GP1661** | ∆*ymdB*  SinR: W104L | Structured colony without wrinkles. | Weak structured pellicle. | - | - |
| **GP1561**  **(NCIB3610,wild type)** | P_hag_-cfp  P_tapA_-yfp | Structured colony with a rough surface and wrinkles. | Robust structured pellicle. | Motility genes expression of the inner region of the colony. Matrix genes expression of the whole colony. | Most cells showed motility genes or no expression of reporter genes. A small subpopulation of cells showed matrix genes expression. Bistable expression. |
| **GP1574** | ∆*ymdB*  P_hag_-cfp  P_tapA_-yfp | Smooth, shiny and unstructured colony. | No pellicle. | Motility genes expression of the whole colony. | Small cells, which showed motility genes expression. |
| **GP1649** | ∆*ymdB*  P_hag_-cfp  P_tapA_-yfp  SinR: W104R | Structured colony with a rough surface, no wrinkles. | Robust structured pellicle | Strong expression of matrix genes and weak expression of motility genes. | Cells showed either matrix genes expression, motility genes expression, or no expression of reporter genes. Bistable expression. |
| **GP1650** | ∆*ymdB*  P_hag_-cfp  P_tapA_-yfp  SinR: A85T | Structured colony, weaker than the wild type. | Stable but unstructured pellicle | Motility genes expression of the inner region of the colony. Matrix genes expression of the whole colony. | Small cells showed motility genes expression, long cells showed either matrix genes or no expression. Bistable expression. |
| **GP1805** | ∆*ymdB*  P_hag_-cfp  P_tapA_-yfp  SinR: L99S | Small colony with a rough and structured surface. | Robust structured pellicle | Strong expression of Matrix genes and weak expression of motility genes. | Highly elongated cells as cell chains. Cells showed strong expression of matrix genes and low expression of motility genes. Some parts of cell chains expressed neither matrix nor motility genes. No bistable expression pattern. |
| **GP1827** | ∆*ymdB*  P_hag_-cfp  P_tapA_-yfp  SinR: K28T | Small colonies with a rough and structured surface. | - | Strong expression of matrix genes and weak expression of motility genes. | - |
