# Supplementary figures and images for: Selective Pressure for Biofilm Formation in Bacillus subtilis: Differential Effect of Mutations in the Master Regulator SinR on Bistability
Source: mBio. 2018 Sep 4;9(5):e01464-18. doi: 10.1128/mBio.01464-18 (PMC6123443; doi:10.1128/mBio.01464-18)

## Slide 1
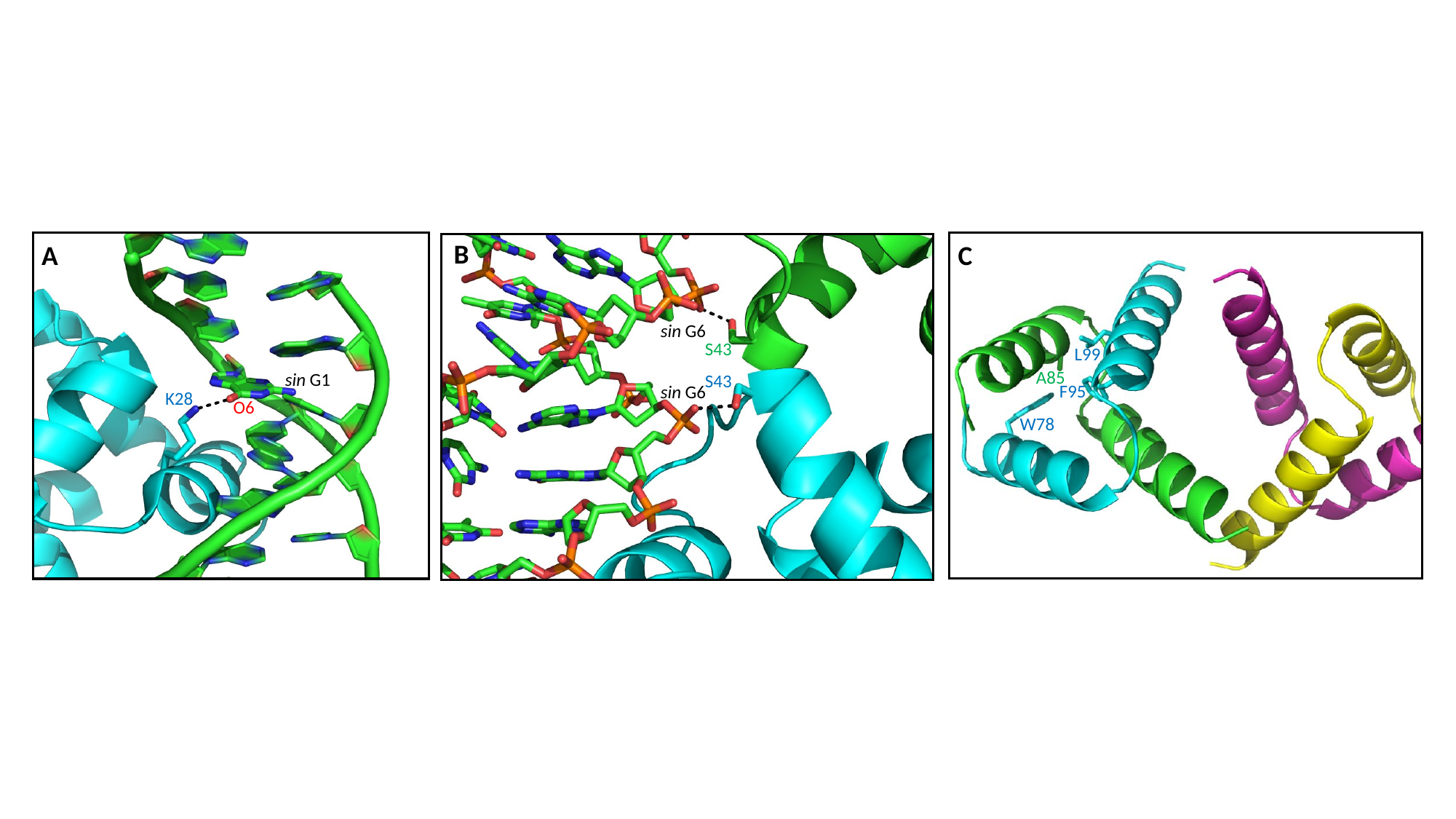

B
sin G6
S43
S43
sin G6
A
sin G1
K28
O6
C
L99
A85
F95
W78

Supplement: FIG S1 [file mbo004184042sf1.ppt]

## Slide 1
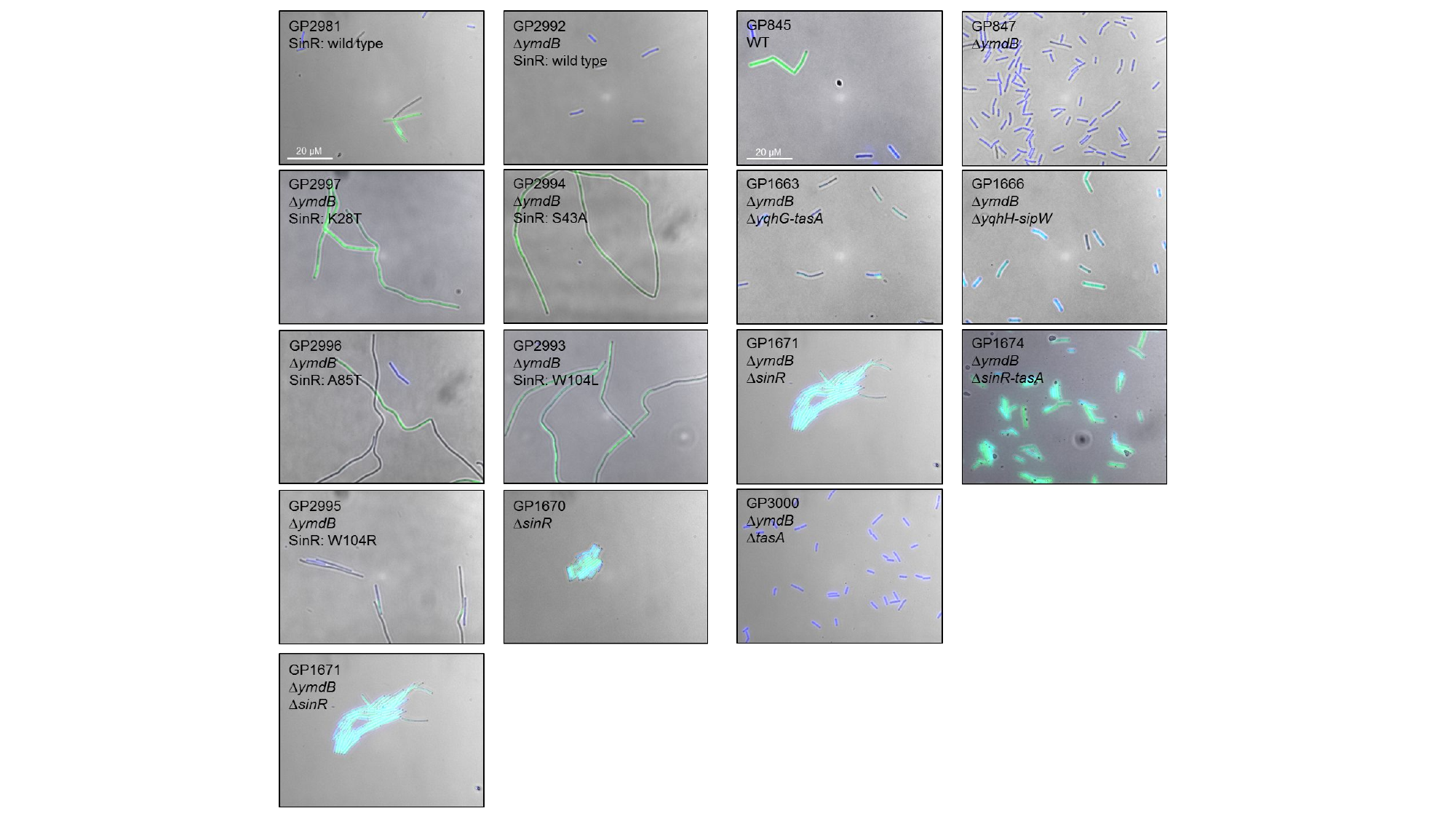

Supplement: FIG S2 [file mbo004184042sf2.ppt]
