# Supplementary material for: Selective Pressure for Biofilm Formation in Bacillus subtilis: Differential Effect of Mutations in the Master Regulator SinR on Bistability
Source: mBio. 2018 Sep 4;9(5):e01464-18. doi: 10.1128/mBio.01464-18 (PMC6123443; doi:10.1128/mBio.01464-18)
Supplement: TABLE S2 [file mbo004184042st2.docx]

**Table S2A. Bacterial strains used in this study.**

| **Strain** | **Genotype** | **Reference/ Construction** | **Remarks** | |  |
| --- | --- | --- | --- | --- | --- |
| ***E. coli*** |  |  |  |  |  |
| BL21(DE3) | fhuA2 [lon] ompT gal (λ DE3) [dcm] ∆hsdS λ DE3 = λ sBamHIo ∆EcoRI-B int::(lacI::PlacUV5::T7 gene1) i21 ∆nin5 | 1 |  |  |  |
| XL1-Blue | *rec*A1 *end*A1 *gyr*A96 *thi*-1 *hsd*R17 *sup*E44 *rel*A1 *lac* | Stratagene |  | | |
| ***B. subtilis*** |  |  |  | | |
| 168 | *trpC2* | Laboratory collection |  | |  |
| 8G5 | *trpC2 tyr-1 his ade met rib ura nic ΔsinR*::*tetR* | Oscar Kuipers |  | |  |
| NCIB3610 | Undomesticated wild type strain | Laboratory collection |  | |  |
| BP494 | *trpC2 bglS*::*(hag-cfp aphA3)* | Paola Bisicchia |  | |  |
| DL382 | *amyE::p(tapA-yfp spc)* | Daniel Lopez |  | |  |
| TMB079 | *ΔsinR::spc* | 2 |  | |  |
| GP583 | *trpC2 ∆ymdB::spc* | 3 |  | |  |
| GP736 | *trpC2 ΔsinR::tet* | 8G5 → 168 |  | |  |
| GP845 | *trpC2* *bglS::(hag-cfp aphA3)* *lacA:: p(tapA-yfp ermC)* | 3 |  | |  |
| GP846 | *amyE::p(tapA-yfp spc)* *ΔymdB::cat* | GP922 → DL382* |  | |  |
| GP847 | *trpC2* *bglS::(hag-cfp aphA3)* *lacA:: p(tapA-yfp ermC) ΔymdB::spc* | 3 |  | |  |
| GP902 | *trpC2 ∆hag::tet* | LFH → 168 |  | |  |
| GP921 | *∆ymdB::spc* | GP583 → NCIB3610* |  | |  |
| GP922 | *trpC2 ∆ymdB::cat* | 3 |  | |  |
| GP1561 | *amyE::p(tapA-yfp spc)* *bglS::(hag-cfp aphA3)* | BP494 → DL382* |  | |  |
| GP1571 | *trpC2 ∆tasA::cat* | LFH → 168 |  | |  |
| GP1574 | *∆ymdB::cat amyE::p(tapA-yfp spc) bglS::(hag-cfp aphA3)* | BP494 → GP846* |  | |  |
| GP1649 | *∆ymdB::cat amyE::p(tapA-yfp spc) bglS::(hag-cfp aphA3) sinR T310A* | GP1574 suppressor mutant | SinR: W104R | |  |
| GP1650 | *∆ymdB::cat amyE::p(tapA-yfp spc) bglS::(hag-cfp aphA3) sinR G253A* | GP1574 suppressor mutant | SinR: A85T | |  |
| GP1657 | ∆*ymdB*::*spc sinR* T127G | GP921 suppressor mutant | SinR: S43A | |  |
| GP1658 | ∆*ymdB*::*spc sinR* G310C | GP921 suppressor mutant | SinR: W104R | |  |
| GP1661 | ∆*ymdB*::*spc sinR* G311T | GP921 suppressor mutant | SinR: W104L | |  |
| GP1663 | *trpC2* *bglS::(hag-cfp aphA3)* *lacA:: p(tapA-yfp ermC)*∆*ymdB::spc* ∆*yqhG-tasA* | GP847 suppressor mutant |  | |  |
| GP1664 | *trpC2* *bglS::(hag-cfp aphA3)* *lacA:: p(tapA-yfp ermC)* ∆*ymdB::spc sinR* A23C | GP847 suppressor mutant | SinR: Gln8Pro | |  |
| GP1665 | *trpC2* *bglS::(hag-cfp aphA3)* *lacA:: p(tapA-yfp ermC)* ∆*ymdB::spc sinR* G311T | GP847 suppressor mutant | SinR: W104L | |  |
| GP1666 | *trpC2* *bglS::(hag-cfp aphA3)* *lacA:: p(tapA-yfp ermC)* ∆*ymdB::spc* ∆*yqhH-sipW* | GP847 suppressor mutant |  | |  |
| GP1667 | *amyE::p(tapA-yfp spc)* ∆*ymdB::cat sinR G312T* | GP846 suppressor mutant | SinR: W104C | |  |
| GP1668 | *amyE::p(tapA-yfp spc)* ∆*ymdB::cat sinR* C272T | GP846 suppressor mutant | SinR: S91L | |  |
| GP1669 | *amyE::p(tapA-yfp spc)* ∆*ymdB::cat sinR* C126T | GP846 suppressor mutant | SinR: P42P | |  |
| GP1670 | *trpC2 ∆sinR::tet bglS::(hag-cfp aphA3) lacA:: p(tapA-yfp ermC)* | GP736 🡪 GP845 |  | |  |
| GP1671 | *trpC2 ∆sinR::tet bglS::(hag-cfp aphA3) lacA:: p(tapA-yfp ermC) ∆ymdB::spc* | GP736 🡪 GP847 |  | |  |
| GP1672 | *trpC2 ∆sinR-tasA*::*cat* | LFH 🡪 168 |  | |  |
| GP1674 | *trpC2 ∆sinR-tasA::cat bglS::(hag-cfp aphA3) lacA:: p(tapA-yfp ermC) ∆ymdB::spc* | GP1672 🡪 GP847 |  | |  |
| GP1805 | *∆ymdB::cat amyE::p(tapA-yfp spc) bglS::(hag-cfp aphA3) sinR T296C* | GP1574 suppressor mutant | SinR: L99S | |  |
| GP1818 | ∆ymdB::cat amyE::p(tapA-yfp spc) bglS::(hag cfp aphA3) ∆sinR::tet | GP736 🡪 GP1574* |  | |  |
| GP1827 | *∆ymdB::cat amyE::p(tapA-yfp spc) bglS::(hag-cfp aphA3) sinR A83C* | GP1574 suppressor mutant | SinR K28T | |  |
| GP2124 | *trpC2 sinR-tet* | LFH → 168 |  | |  |
| GP2130 | *trpC2 bglS::(hag-cfp aphA3) lacA:: p(tapA-yfp ermC) Δhag::tet* | GP902 → GP845 |  | |  |
| GP2551 | *trpC2 bglS::(hag-cfp aphA3) lacA:: p(tapA-yfp ermC) Δhag::tet ΔymdB::spc* | GP583 → GP2130 |  | |  |
| GP2554 | *comIQ12L ∆ymdB::cat sinR::spc* | PCR Pro. CD145/146 TMB079 →GP2559 | DK1042 | |  |
| GP2559 | *comIQ12L ∆ymdB::cat* | PCR Pro. SHU63/NP61 GP922 → DK1042 |  | |  |
| GP2560 | *comIQ12L sinR-tet* | PCR Pro. CD145/146 GP2124 → DK1042 | DK1042 | |  |
| GP2570 | *comIQ12L ∆sinR::spc* | PCR Pro. CD145/146 TMB079 → DK1042 | DK1042 | |  |
| GP2976 | *comIQ12L sinR-tet* | LFH → DK1042 (GP1650 up-element template) | DK1042, SinR: A85T | |  |
| GP2977 | *comIQ12L sinR-tet* | LFH → DK1042 (GP1657 up-element template) | DK1042, SinR: S43A | |  |
| GP2978 | *comIQ12L sinR-tet* | LFH → DK1042 (GP1658 up-element template) | DK1042, SinR: W104R | |  |
| GP2979 | *comIQ12L sinR-tet* | LFH → DK1042 (GP1665 up-element template) | DK1042 ,SinR: W104L | |  |
| GP2980 | *comIQ12L sinR-tet* | LFH → DK1042 (GP1827 up-element template) | DK1042, SinR: K28T | |  |
| GP2981 | *trpC2 bglS::(hag-cfp aphA3) lacA:: p(tapA-yfp ermC) sinR-tet* | LFH →GP845 |  | |  |
| GP2987 | *comIQ12L sinR-tet ymdB::cat* | PCR CD145/146 GP2976 → GP2559 | DK1042, SinR: A85T | |  |
| GP2988 | *comIQ12L sinR-tet ymdB::cat* | PCR CD145/146 GP2977 → GP2559 | DK1042, SinR: S43A | |  |
| GP2989 | *comIQ12L sinR-tet ymdB::cat* | PCR CD145/146 GP2978 → GP2559 | DK1042, SinR: W104R | |  |
| GP2990 | *comIQ12L sinR-tet ymdB::cat* | PCR CD145/146 GP2979 → GP2559 | DK1042 ,SinR: W104L | |  |
| GP2991 | *comIQ12L sinR-tet ymdB::cat* | PCR CD145/146 GP2980 → GP2559 | DK1042, SinR: K28T | |  |
| GP2992 | *trpC2 bglS::(hag-cfp aphA3) lacA:: p(tapA-yfp ermC) sinR-tet ymdB::spc* | PCR CD145/146 GP2560 → GP847 |  | |  |
| GP2993 | *trpC2 bglS::(hag-cfp aphA3) lacA:: p(tapA-yfp ermC) sinR-tet sinR G253A ymdB::spc* | PCR CD145/146 GP2976 → GP847 | SinR: A85T | |  |
| GP2994 | *trpC2 bglS::(hag-cfp aphA3) lacA:: p(tapA-yfp ermC) sinR-tet sinR T127G ymdB::spc* | PCR CD145/146 GP2977 → GP847 | SinR: S43A | |  |
| GP2995 | *trpC2 bglS::(hag-cfp aphA3) lacA:: p(tapA-yfp ermC) sinR-tet sinR G310C ymdB::spc* | PCR CD145/146 GP2978 → GP847 | SinR: W104R | |  |
| GP2996 | *trpC2 bglS::(hag-cfp aphA3) lacA:: p(tapA-yfp ermC) sinR-tet sinR G311T ymdB::spc* | PCR CD145/146 GP2979 → GP847 | SinR: W104L | |  |
| GP2997 | *trpC2 bglS::(hag-cfp aphA3) lacA:: p(tapA-yfp ermC) sinR-tet sinR A83C ymdB::spc* | PCR CD145/146 GP2980 → GP847 | SinR: K28T | |  |
| GP2998 | *comIQ12L sinR-tet ymdB::cat* | PCR CD145/146 GP2560 → GP2559 | DK1042 | |  |
| GP3000 | *trpC2* *bglS::(hag-cfp aphA3)* *lacA:: p(tapA-yfp ermC) ΔymdB::spc ΔtasA::cat* | GP1571 🡪 GP847 |  | |  |

Arrows indicate construction by transformation. *, Construction by phage transduction. LFH, long flanking homology PCR.

References

1. F.W. Studier and B.A. Moffatt, J Mol Biol 189:113-130, 1986.
2. S. Jordan, E. Rietkötter, M.A. Strauch, F. Kalamorz, B.G. Butcher, J.D. Helmann, and, T. Mascher, Microbiology 153:2530–2540, 2007.
3. C. Diethmaier, N. Pietack, K. Gunka, C. Wrede, M. Lehnik-Habrink, C. Herzberg, S. Hübner, and J. Stülke, J Bacteriol 193:5997-6007, 2011.

**Table S2B. Oligonucleotides used in this study**

| **Oligonucleotide** | **Sequence** | **Restriction sites** |
| --- | --- | --- |
| **Cloning of *sinR* variants into the pET24a vector** | |  |
| G8 | GATATA**CATATG**ATTGGCCAGCGTATTAAAC | *Nde*I |
| G10 | CGTATA**GAATTC**TCACTCCTCTTTTTGGGATTTTC | *Eco*RI |
| **Sequencing of *sinIR* regions** |  |  |
| ***sinIR region*** |  |  |
| KK47 | CAACCAAAGATATCAGTTGAAATTGAAAATGGCG |  |
| JG157 | GTGGAAGTGGGAGCTTCATAAGCTTG |  |
| ***sinIR* in GP1663 ∆*yqhG-tasA*** |  |  |
| ML26 | gaggaacatgggcagcat |  |
| CD145 | GCTGCCAAATCGTCGATCAAGGC |  |
| ***sinIR* in GP1666 ∆*yqhH-sipW*** |  |  |
| KK52 | ATGAATACAGAAATGATCTACGATGCAAAATGGCC |  |
| CD226 | AAA**GAATTC**CTCAGAGTTAAATGGTATTGCTTCACT | *Eco*RI |
| **Construction of mutants** | |  |
| **Amplification of up- and downstream element of *hag*** | |  |
| CD47 | GGGTGAAAATACAATATACTCCGTCAC |  |
| CD48 | *CCTATCACCTCAAATGGTTCGCTG*TGTTTTGTTCCTCCCTGAATATGTTG |  |
| CD49 | *CGAGCGCCTACGAGGAATTTGTATCG*CAAGCAAACCAACAGCCGCAAAACG |  |
| CD50 | GTTTTTGCTGTTGTTTCGCCAGGCG |  |
| **Amplification of up- and downstream elements of *sinR, sinR-tasA, tasA,* and for *sinR-tet* fusions** | |  |
| sinR_up_fwd | GCCAAAAGACCTAGATGGTG |  |
| sinR_up_rev | CCTATCACCTCAAATGGTTCGCTGATGTCATCACCTTCCTTGTG |  |
| CD141 | *CGAGCGCCTACGAGGAATTTGTATCG*CCGGGGTATCGAAAAAACAATTTCGTG |  |
| CD142 | CTTCAGTTGTAAACCTGGCAACAGG |  |
| JG153 | CGGCGATTCACTTTATAAAATTGAGACCAAG |  |
| JG154 | *CCTATCACCTCAAATGGTTCGCTG*GCTAATCCTAGTGCTGCAGAAGCAAC |  |
| JG155 | *CGAGCGCCTACGAGGAATTTGTATCG*CGGCTTGACAATCAAAAAGGACCATACTG |  |
| JG156 | CAGGCGCTGAAAACCTTGTATCAACC |  |
| JK155 | *CCTATCACCTCAAATGGTTCGCTG*GCACTACTCCTCTTTTTGGGATTTTC |  |
| JK156 | *CGAGCGCCTACGAGGAATTTGTATCG*CTGAGCAGAGGCACTAACTCC |  |
| **Amplification of *sinR-tet* cassettes** | |  |
| CD145 | GCTGCCAAATCGTCGATCAAGGC |  |
| CD146 | GACGATCAGCAGCGCCATTAGAG |  |
| **Amplification of *ymdB::cat* cassette** | |  |
| SHU63 | CCGTGCGAAAGAAGAGGCGG |  |
| NP61 | AGTATTGGTACACACATGAGATTTTCCTGTTAG |  |
| **Amplification of chloramphenicol resistance cassette** | |  |
| cat-fwd (kan) | *CAGCGAACCATTTGAGGTGATAGG*CGGCAATAGTTACCCTTATTATCAAG |  |
| cat-rev (kan) | *CGATACAAATTCCTCGTAGGCGCTCGG*CCAGCGTGGACCGGCGAGGCTAGTTACCC |  |
| **Amplification of tetracyline resistance cassette** |  |  |
| tc-fwd2(kan) | *CAGCGAACCATTTGAGGTGATAGG*GCTTATCAACGTAGTAAGCGTGG |  |
| tc-rev(kan) | *CGATACAAATTCCTCGTAGGCGCTCGG*GAACTCTCTCCCAAAGTTGATCCC |  |
| **SinR box cassette** |  |  |
| FAM1721 | 5‘-FAM-ATTGTTCTCTAAAGAGAACTT |  |
| C-1723 | AAGTTCTCTTTAGAGAACAAT |  |

**Bold**, restriction sites

*Italic*, Kanamycin overhangs for LFH fusion PCR

FAM, flourescin

**Table S2C. Plasmids used in this study.**

| **Plasmid** | **Description** | **Construction/ Reference** |  |
| --- | --- | --- | --- |
| pET24a | Vector for in vitro expression *via* T7 promoter; kanamycin resistance; restriction enzyme cloning. | Novagen |  |
| pDG1514 | Vector for tetracycline resistance cassette for LFH of *B. subtilis* | 1 |  |
| pC2 | *sinI* in the expression vector pET24a | 2 |  |
| pC5 | *sinR* in the expression vector pET24a | 2 |  |
| pGEM-cat | Vector for chloramphenicol resistance cassette for LFH of *B. subtilis* | 3 |  |
| pGP1948 | pET24a/ *Nde*I/*Eco*RI | PCR Prod. *sinR G253A*, G8/G10/ *Nde*I/*Eco*RI | amplified using GP1650 as template |
| pGP1949 | pET24a/ *Nde*I/*Eco*RI | PCR Prod. *sinR* T127G, G8/G10/ *Nde*I/*Eco*RI | amplified using GP1657 as template |
| pGP1950 | pET24a/ *Nde*I/*Eco*RI | PCR Prod. *sinR* G310C, G8/G10/ *Nde*I/*Eco*RI | amplified using GP1658 as template |
| pGP2302 | pET24a/ *Nde*I/*Eco*RI | PCR Prod. *sinR* G311T, G8/G10/ *Nde*I/*Eco*RI | amplified using GP1665 as template |
| pGP2304 | pET24a/ *Nde*I/*Eco*RI | PCR Prod. *sinR A83C*, G8/G10/ *Nde*I/*`*RI | amplified using GP1827 as template |

References

1. A.M. Guérout-Fleury, K. Shazand, N. Frandsen, and P. Stragier, Gene 167:335–336, 1995.
2. J.A. Newman, C. Rodrigues, and R.J. Lewis RJ, J Biol Chem 288:10766–10778, 2013.
3. **P. Youngman,** p. 221-266 in C.R. Harwood and S.M. Cutting, ed., *Molecular biological methods for* Bacillus 1990.
