# Supplementary material for: Selective Pressure for Biofilm Formation in Bacillus subtilis: Differential Effect of Mutations in the Master Regulator SinR on Bistability
Source: mBio. 2018 Sep 4;9(5):e01464-18. doi: 10.1128/mBio.01464-18 (PMC6123443; doi:10.1128/mBio.01464-18)
Supplement: FIG S3 [file mbo004184042sf3.ppt]

## Slide 1
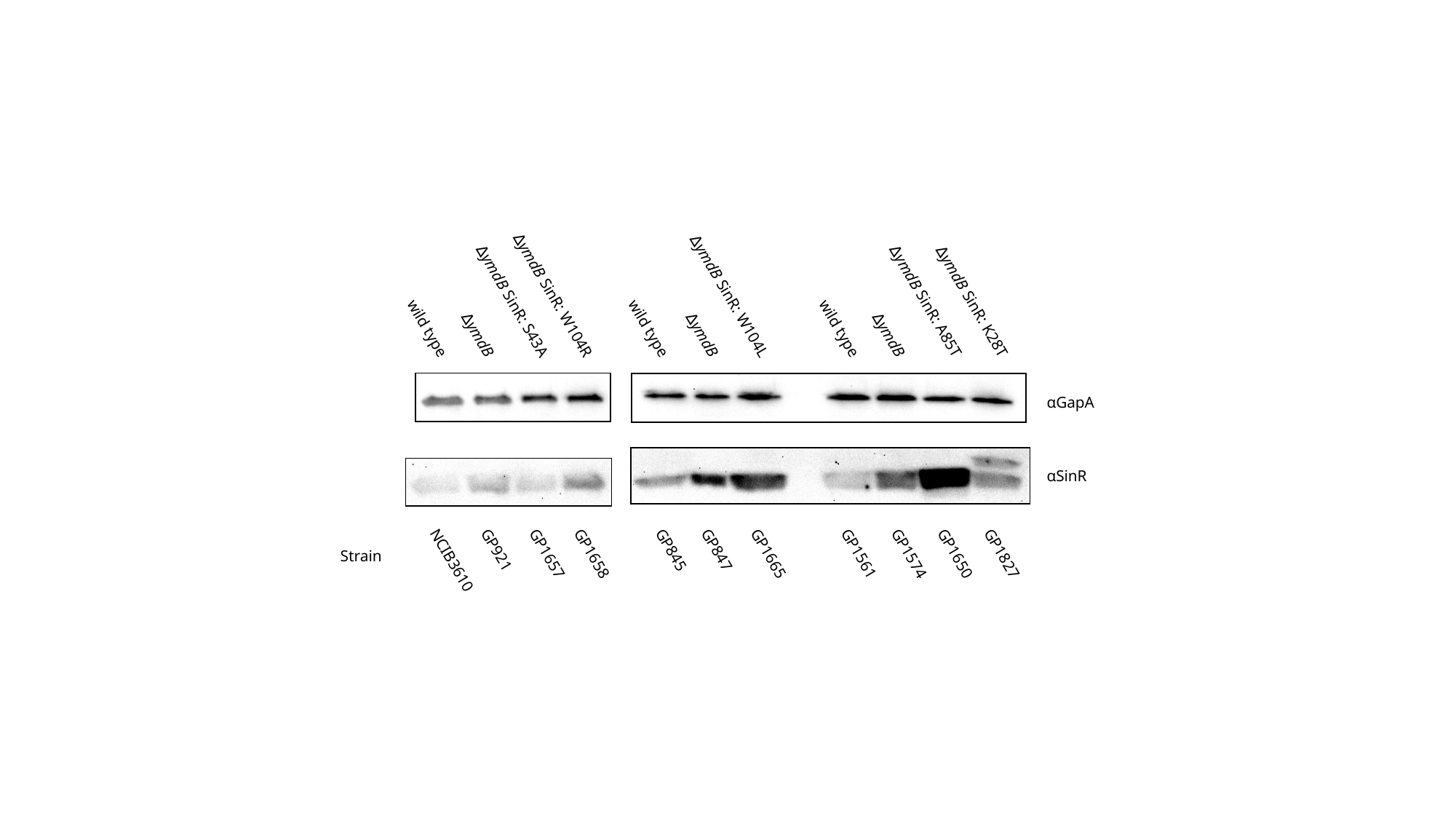

∆ymdB SinR: W104R
∆ymdB SinR: W104L
wild type
∆ymdB
∆ymdB SinR: S43A
wild type
∆ymdB
wild type
∆ymdB
∆ymdB SinR: A85T
∆ymdB SinR: K28T
αGapA
αSinR
Strain
GP921
GP1657
GP1658
GP845
GP847
GP1665
GP1561
GP1574
GP1650
GP1827
NCIB3610
